# Supplementary material for: The Histopathological “Placentitis Triad” Is Specific for SARS-CoV-2 Infection, and Its Acute Presentation Can Be Associated with Poor Fetal Outcome
Source: Life (Basel). 2023 Feb 9;13(2):479. doi: 10.3390/life13020479 (PMC9963180; doi:10.3390/life13020479)
Supplement: Supplementary file 1 [file life-13-00479-s001.zip › life-2145098-supplementary.pdf]

**Supplementary Table S1 :** Summary of the placental lesions in reported cases of *SARS-CoV-2* infection in the literature [3-6, 8-10, 12-17, 20-22, 25-46].

*Abbreviations : AI acute inflammation, CA chorioamniotis, CCA chronic chorioamniotis, CI chronic inflammation, CHI chronic histiocytic intervillositis, CS cesarean section, DIC disseminated intravascular coagulation, ECMO extracorporeal membrane oxygenation, FVM fetal vascular malperfusion, WG gestational week, IUFD intra uterine fetal death, IVT intervillous thrombosis, EFD excess fibrin deposition, MVM maternal vascular malperfusion, RDS respiratory distress syndrome.*

| Study | Meta analysis | number of cases | clinical data | Placental weight | histological data |
|---|---|---|---|---|---|
| Girolamo 2021 | yes, 57 studies | 895 (with complete histopathological analysis of the placenta) | | | 30,7% MVM, 26,9% FVM, 32,7% EFD, 22,6% AI, 26,2% CI, 17,5% normal, 14,5% IVT |
| Wong 2021 | yes, 29 studies (20 not described in Girolamo study) | 328 | | | 37,8% MVM, 9,2% FVM, 43,2% EFD, 34,7% inflammation, 29,4% intervillositis, 14,7% villitis, 35,3% CA, 35,1% infarction, 5,4% thrombosis |
| Sharp 2020 | yes, 50 studies (14 neither described in Girolamo or Wong studies) | 150 | | | 46% MVM, 35,3% FVM, 8,7% villitis, 5,3% intervillositis, 6% CA, 30% EFD |

| | | | | | |
|---|---|---|---|---|---|
| Bouachba 2021 | no | 5 | 27,6 WG (average), 3 stillbirth, 2 extreme premature births at 26 WG | < 10th percentile for all | CHI for all, EFD for all, IVT for 4/5 |
| Giordano 2021 | no | 5 | 38,8 WG (average), good extra uterine adaptation for all | 2 cases <10th percentile | 3/5 FVM, 3/5 IVT, 2/5 villitis, 1/5 CA |
| Babal 2021 | no | 1 | 38 WG ; fetal death, maternal Covid-19 infection 22 days before | Normal weight | Intervillous EFD, villitis, infarction, villous necrosis, description of intervillositis |
| Favre 2021 | no | 2 | 29 and 32 WG ; diminution of fetal movements, maternal Covid-19 infection 5 days before, CS, severe brain injury | Normal weight | EFD 80%, extensive and focal chronic intervillositis, infarction |
| Mao 2021 | no | 1 | 20 WG ; fetal death, maternal Covid-19 infection 2 days before | < 10th percentile | EFD and chronic intervillositis at 80% |
| Moresi 2021 | no | 106 | 39,1 WG ; maternal Covid-19 infection at 37,1 WG (average) ; 8,5% of neonatal care | 19,2th percentile (average) | MVM, 43,3% extensive fibrin deposition, 7,5% chronic intervillositis |

| | | | | | |
|---|---|---|---|---|---|
| Celik 2021 | no | 20 | 2 groups : low / asymptomatic, mild / severe maternal symptoms | | 57% histological anomalies in mild symptoms, 100% in severe symptoms ; 4 cases of perivillous fibrin, 3 cases of intervillositis, 1 case of intervillositis with deciduitis (with CMV infection) |
| Briana 2021 | no | 40, 18 with histological data | 39 WG (average) | | MVM, decidual arteriopathy, various degree of intervillous maternal thrombosis, fibrinoid deposition, mild intervillositis, lymphoplasmocytic deciduitis |
| Rosner 2021 | no | 1 | 27 WG ; severe RDS, ECMO, CS | | MVM, chorangioma (1 cm), CCA |
| Thomas 2021 | no | 197 | 1 miscarriage, 1 fetal death, 1 neonatal death | | 1,02% intervillositis |
| Dumont | no | 1 | 29 WG ; diminution of fetal movements, CS | | Placentitis triad = intervillous fibrin deposition, ischemic necrosis of villi, histiocytic intervillositis |

| | | | | | |
|---|---|---|---|---|---|
| Laresgoiti 2021 | no | 66 | 2 miscarriages, 2 fetaldeath | 19,44th mean percentile | 90,91% of placental fibrinoid |
| Meyer 2021 | no | 61 | 5 IUFD | 25% small weight for gestational age | 77% with almost one sign of MVM, 59% perivillous EFD |
| Brien 2021 | no | 66 | | 15 cases < 10th percentile | 80% with histological anomalies ; 20 cases with MVM, 18 cases of FVM, 32 with excess fibrin |
| Marinho 2021 | no | 1 | 34 WG, fetal death | 80th percentile | acute and chronic intervillositis, intervillous microabscesses, chronic and proliferative villitis, acute deciduitis, severe MVM with extensive and diffuse intervillous / pervillous fibrin deposition, acute FVM |
| Mai 2021 | no | 1 | Morbid obesity | | FVM |

| | | | | | |
|---|---|---|---|---|---|
| Husen 2021 | no | 39 | 30,6% premature birth | | 62% with almost one histologic anomaly ; 4 cases with "SARS-Cov-2 placental signature" = CHI, EFD, syncytiotrophoblast necrosis, CD20+ ; 9 cases with perivillous fibrin |
| Schwartz 2021 | no | 1 | 31 WG, DIC, CS | Normal weight | CHI, with neutrophils, EFD on 30% of the placenta |
| Jin 2021 | no | 2 | 38 and 39 WG | | Acute necrotizing deciduitis, intervillous EFD, acute intervillositis |
| Watkins 2021 | no | 7 | 1 miscarriage | | All with placentitis = CHI, perivillous fibrin deposition, trophoblast necrosis ; 3 cases with FVM |
| Biringer 2021 | no | 1 | Sudden preterm fetal death | | Inflammation with fetal inflammatory response syndrome |
| Mourad 2021 | no | 66 | 59 cases with asymptomatic / mild maternal symptoms, 7 with severe maternal disease | | MVM, FVM, acute and chronic inflammation |

| | | | | | |
|---|---|---|---|---|---|
| Popescu 2021 | no | 1 | 25 WG, fetal death with hydrops, 7 weeks after maternal recovery from a mild SARS-CoV-2 infection | > 90th percentile | Fetal systemic thrombosis, acute subchorionitis, FVM, intervillous thrombi, perivillous EFD, intervillositis with neutrophils and monocytes |
| Roberts 2021 | no | 1 | Preterm birth | small for gestational age | Extensive intervillous histiocytosis, syncytiotrophoblast karyorrhexis, diffuse intervillous EFD |
| Argueta 2021 | no | 54 | 2 IUFD, 1 premature birth | | 31% FVM, 19% MVM, 7% both FVM and MVM, CHI in trophoblast virus context |
| Marton 2021 | no | 1 | diminution of fetal movements at 25 WG, then death | | CHI, trophoblast necrosis, EFD on 90% of the placenta |
| Resta 2021 | no | 1 | gemellar gestation, 30 WG, perinatal death of one fetus after few minutes | Normal weight | On both sides, large EFD, CHI |

| | | | | | |
|---|---|---|---|---|---|
| Jang 2021 | no | 7 | 14,5 WG (mean) for Covid diagnosis, 38,4 WG (mean) for delivery | | 1 case with MVM with EFD, 4 cases of FVM, 2 cases with inflammation |
| Garrido 2021 | no | 198, with 9 cases Covid-positive | | | for the 9 cases, villous trophoblast necrosis, intervillous space collapse, mixed intervillous inflammatory infiltrate, EFD |
| JR He 2021 | no | 1 | 37 WG ; Covid infection at 7 WG | Normal weight | Placental insufficiency not linked to Covid |
| Baral 2021 | no | 1 | IUFD | | Widespread villous infarction, decidual arteriopathy with thrombosis and chorioamnionitis |
| Zhang 2021 | no | 101 | | | noincrease of placentalpathologyfeatures |
| Zaigham 2021 | no | 1 | 34 WG, diminution of fetal movements and abdominal pain, CS, fetal death | | EFD, acute intervillositis, degeneration of villous trophoblast layer on 50% of the placenta |

| | | | | | |
|---|---|---|---|---|---|
| Poisson 2021 | no | 1 | 35 WG, IUFD, covid infection at 32 WG | | Acute chorionitis, 75% diffuse infarction, villous necrosis, extensive FVM |
| Dubucs 2022 | no | 50 | 1-3 weeks after positive test | | 10 with trophoblast necrosis, fibrinous deposits, intervillositis and extensive hemorrhagic changes, comprising 5 cases with IUFD |
